# Supplementary material for: Hierarchical entanglement shells of multichannel Kondo clouds
Source: Nat Commun. 2023 Jun 14;14:3521. doi: 10.1038/s41467-023-39234-6 (PMC10267179; doi:10.1038/s41467-023-39234-6)
Supplement: Supplementary file 1 — Supplementary Information [file 41467_2023_39234_MOESM1_ESM.pdf]

Supplementary Information for "Hierarchical entanglement shells of multichannel Kondo clouds "

Jeongmin Shim[1,2,3], Donghoon Kim[1,3], and H.-S. Sim[1,*]
[1] *Department of Physics, Korea Advanced Institute of Science and Technology, Daejeon 34141, Korea*
[2] *Present address: Arnold Sommerfeld Center for Theoretical Physics, Center for NanoScience, and Munich Center for Quantum Science and Technology, Ludwig-Maximilians-Universität München, 80333 Munich, Germany*
[3] *These authors contributed equally: Jeongmin Shim, Donghoon Kim*

This material contains details in the NRG, BCFT, and bosonization calculations and estimate of experimental parameters. Below we set $\hbar \equiv 1$, $v_{\mathrm{F}} = 1$, and $k_{\mathrm{B}} \equiv 1$ somewhere for simplicity.

Supplementary Note 1. DERIVATION OF EQ. (3)

We derive Eq. (3) of the main text. An equivalent proof is found in Supplementary Materials (see Sec. III) of Ref. [S8]. We consider the kCK model affected by the LSB, i.e., $H = H_{k\mathrm{CK}} + H_{\mathrm{LSB}}$ [See Eqs. (1) and (4) of the main text]. In this case, the SU(2) symmetry is broken and the ground state becomes nondegenerate. The nondegenerate ground state $|E\rangle$ is written in a Schmidt decomposed form,

$$|E\rangle = \sum_{i=1,2} \sqrt{p_i}|\phi_i\rangle_A \otimes |\psi_i\rangle_B, \tag{S1}$$

where A and B denote the impurity and its environment, respectively, and $p_1 + p_2 = 1$. Since the Hilbert-space dimension of the impurity is 2, the state is expressed by two orthonormal bases $\{|\phi_i\rangle_A\}_{i=1,2}$ and $\{|\psi_i\rangle_B\}_{i=1,2}$. In the basis $\{|\phi_1\rangle_A|\psi_1\rangle_B, |\phi_1\rangle_A|\psi_2\rangle_B, |\phi_2\rangle_A|\psi_1\rangle_B, |\phi_2\rangle_A|\psi_2\rangle_B\}$, the density matrix and its partial transpose are written as

$$\rho = |E\rangle\langle E| \doteq \begin{pmatrix} p_1 & 0 & 0 & \sqrt{p_1 p_2} \\ 0 & 0 & 0 & 0 \\ 0 & 0 & 0 & 0 \\ \sqrt{p_1 p_2} & 0 & 0 & p_2 \end{pmatrix}, \qquad \rho^{\mathrm{T}_A} \doteq \begin{pmatrix} p_1 & 0 & 0 & 0 \\ 0 & 0 & \sqrt{p_1 p_2} & 0 \\ 0 & \sqrt{p_1 p_2} & 0 & 0 \\ 0 & 0 & 0 & p_2 \end{pmatrix}. \tag{S2}$$

Here, T_A is the partial transpose on the impurity. Then the singular values of ρ^{T_A} are p_1, p_2, and two $\sqrt{p_1 p_2}$, so the trace norm $\|\rho^{\mathrm{T}_A}\|_1$, the sum of these singular values, is $p_1 + p_2 + 2\sqrt{p_1 p_2} = 1 + 2\sqrt{p_1 p_2}$. Therefore, it leads to the entanglement negativity between the impurity and its enviroment

$$\mathcal{N} = \|\rho^{\mathrm{T}_A}\|_1 - 1 = 2\sqrt{p_1 p_2}, \tag{S3}$$

which is solely determined by p_1 and p_2.

We now calculate p_1 and p_2. From Eq. (S1), the reduced density matrix of the impurity system is derived as

$$\rho_A = \mathrm{Tr}_B[|E\rangle\langle E|] = \sum_{i=1,2} p_i |\phi_i\rangle_A\langle\phi_i|. \tag{S4}$$

Namely, $\{|\phi_i\rangle_A\}_{i=1,2}$ and $\{p_i\}_{i=1,2}$ are the eigenvectors and eigenvalues of ρ_A. Since ρ_A is an operator on the impurity that has two levels, it is written as a linear combination of the identity operator $\mathbb{I}$ and Pauli matrices $\sigma_{x,y,z}$,

$$\rho_A = a_0\mathbb{I} + a_1\sigma_x + a_2\sigma_y + a_3\sigma_z. \tag{S5}$$

Using $\mathrm{Tr}[\rho_A] = 1$ and $\mathrm{Tr}[\rho_A S^{x,y,z}_{\mathrm{imp}}] = \langle E|S^{x,y,z}_{\mathrm{imp}}|E\rangle \equiv \langle S^{x,y,z}_{\mathrm{imp}}\rangle$ ($S^{\alpha}_{\mathrm{imp}} = \hbar\sigma_\alpha/2$ is the impurity spin for $\alpha = x, y, z$), Eq. (S5) becomes

$$\rho_A = \frac{1}{2}\mathbb{I} + \frac{\langle S^x_{\mathrm{imp}}\rangle}{\hbar}\sigma_x + \frac{\langle S^y_{\mathrm{imp}}\rangle}{\hbar}\sigma_y + \frac{\langle S^z_{\mathrm{imp}}\rangle}{\hbar}\sigma_z. \tag{S6}$$

The eigenvalues of ρ_A in Eq. (S6) are

$$p_1 = \frac{1}{2}\left(1 + \frac{2}{\hbar}\sqrt{\langle S^x_{\mathrm{imp}}\rangle^2 + \langle S^y_{\mathrm{imp}}\rangle^2 + \langle S^z_{\mathrm{imp}}\rangle^2}\right), \qquad p_2 = \frac{1}{2}\left(1 - \frac{2}{\hbar}\sqrt{\langle S^x_{\mathrm{imp}}\rangle^2 + \langle S^y_{\mathrm{imp}}\rangle^2 + \langle S^z_{\mathrm{imp}}\rangle^2}\right). \tag{S7}$$

Combining Eqs. (S3) and (S7), we obtain

$$\mathcal{N} = \sqrt{1 - \frac{4}{\hbar^2}(\langle S^x_{\rm imp}\rangle^2 + \langle S^y_{\rm imp}\rangle^2 + \langle S^z_{\rm imp}\rangle^2)}. \tag{S8}$$

Since $\mathbf{M} = \langle E|\mathbf{S}_{\rm imp}|E\rangle = \langle \mathbf{S}_{\rm imp}\rangle = \langle S^x_{\rm imp}\rangle \hat{\mathbf{e}}_x + \langle S^y_{\rm imp}\rangle \hat{\mathbf{e}}_y + \langle S^z_{\rm imp}\rangle \hat{\mathbf{e}}_z$, Eq. (S8) is equal to Eq. (3) of the main text.

Supplementary Note 2. NRG CALCULATION

Our NRG calculation of the entanglement negativity $\mathcal{N}$ is done, using the method developed in Ref. [S1]. Below we describe the Hamiltonian and parameters used in the calculation.

In the total Hamiltonian $H_{k\rm CK} + H_{\rm LSB}$, $H_{k\rm CK}$ describes the kCK model,

$$H_{k\rm CK} = \sum_{j=1}^{k} H_j + \sum_{j=1}^{k} J_j \mathbf{S}_{\rm imp}\cdot\mathbf{S}_j. \tag{S9}$$

$H_j = \frac{D}{2}\sum_{\alpha=\pm}\sum_{\ell=0}\left[\psi^\dagger_{\alpha j}(\ell)\psi_{\alpha j}(\ell+1) + \psi^\dagger_{\alpha j}(\ell+1)\psi_{\alpha j}(\ell)\right]$ is a semi-infinite one-dimensional tight-binding chain Hamiltonian for the jth conduction channel. D is a half band width of the chain. $\psi_{\alpha j}(\ell)$ is an annihilation operator of an electron having spin α at the ℓ-th site of the jth chain. $\alpha = \pm$ represents the eigenspin states of the spin operator S^x in the x direction. The impurity spin $\mathbf{S}_{\rm imp}$ is coupled to the spin $\mathbf{S}_j$ at the 0-th site of the jth chain with the coupling strength J_j. The local spin symmetry breaking perturbation $H_{\rm LSB}$ at nth conduction channel is described by

$$H_{\rm LSB} = \frac{B}{2}\left[\psi^\dagger_{+n}(L)\psi_{+n}(L) - \psi^\dagger_{-n}(L)\psi_{-n}(L)\right]. \tag{S10}$$

This Hamiltonian breaks the SU(2) spin symmetry such that an electron has a different energy depending on the direction of its spin S^x at the L-th site of the nth channel. B is the strength of the symmetry breaking.

To solve the total Hamiltonian $H_{k\rm CK} + H_{\rm LSB}$ in the NRG approach, we obtain the local densities of states (LDOSs) of conduction electrons at the 0-th site of the channels, based on the Hamiltonian $\sum_{j=1}^{k} H_j + H_{\rm LSB}$ of conduction electrons and using the equations of motion for the Green function $G(\epsilon)$. In the $j(\neq n)$-th channel where the local symmetry breaking perturbation is not applied, the Green function of an electron of spin α and energy ϵ at the 0-th site is $G_{\alpha j} = G_0 = 2(\epsilon - i\sqrt{D^2-\epsilon^2})/D^2$ and the LDOS is found as $\nu_{\alpha j} = -\frac{1}{\pi}\mathrm{Im}[G_{\alpha j}(\epsilon)]$. In the nth channel where the local symmetry breaking perturbation is applied, we solve the equations of motion and find that the Green function of an electron of spin $\alpha = \pm$ and energy ϵ at the 0-th site is

$$G_{\alpha n}(\epsilon) = \frac{\sqrt{D^2-\epsilon^2} + (2/\tilde{G} - \epsilon)\tan(L\varphi)}{\sqrt{D^2-\epsilon^2} + (\epsilon - \tilde{G}D^2/2)\tan(L\varphi)}\tilde{G}, \tag{S11}$$

where $\tilde{G}(\epsilon) = [(\epsilon + \alpha B) - (D/2)^2 G_0(\epsilon)]^{-1}$ and $\varphi = \mathrm{atan}(\sqrt{(D/\epsilon)^2 - 1})$. Then the LDOS is obtained as $\nu_{\alpha n} = -\frac{1}{\pi}\mathrm{Im}[G_{\alpha n}(\epsilon)]$. When the half band width D is much larger than temperature T and the strength B of the spin symmetry breaking, the LDOSs approximately follow $\nu_{\alpha j} \sim 1/2D$ for $j \neq n$ and

$$\nu_{\alpha n}(\epsilon) \sim \frac{1}{2D}\left[1 - \alpha(-1)^L \frac{2B}{D}\sin\left(\frac{2\epsilon L}{D}\right)\right]. \tag{S12}$$

We use these approximated LDOSs in the NRG calculation.

We discuss the details [S2, S3] and the parameters of the NRG calculation. We employ the full density matrix NRG [S4, S5] and the interleaved NRG [S6, S7] for spin and channel indices. We set the half band width $D = 1$, the discretization parameter $\Lambda = 10$, and the length of the Wilson chain by 28. We choose the number of kept states by 300 for the 1CK model, 3,000 for 2CK, and 10,000 for 3CK. The result in Fig. 1 of the main text is obtained with the Kondo coupling $J = 0.3D$, the perturbation strength $B = 0.1D$, and the z-averaging of $z = 0$, 1/4, 1/2 and 3/4. The result in Figs. 2 and 3 is obtained with $J = 0.3D$, $B = 0.1D$, and the z-averaging of $z = 0$ and 1/2. The result in Fig. 4 is obtained with $J = 0.4D$, $B = 0.4D$, and the z-averaging of of $z = 0$ and 1/2. We choose the Kondo temperature $T_{\rm K} = \exp(-1/\nu J)$ and the Kondo length $\xi_{\rm K} = \hbar v_{\rm F}/k_{\rm B}T_{\rm K}$ where $\nu = 1/(2D)$. For the Kondo coupling $J = 0.3D$, $T_{\rm K} \sim 1.273\times10^{-3}D$ and $\xi_{\rm K} \sim 4.937\times10^{3}/D$. We set the Planck constant $h = 1$, the Boltzmann constant $k_{\rm B} = 1$, and the Fermi velocity $v_{\rm F} = 1$ in the NRG calculation.

We note that the results of $\rho_n < \mathcal{O}(10^{-5})$ and $\mathcal{O}(10^{-3})$ are not shown in Fig. 3 of the main text, respectively, as the NRG is less accurate at higher T [S2, S3].

Supplementary Note 3. UNIVERSAL SCALING OF KONDO CLOUD

In Supplementary Figs. 1-4, we present the universal scaling of the spatial distribution of Kondo clouds with respect to the Kondo length ξ_K and the crossover length ξ^*.

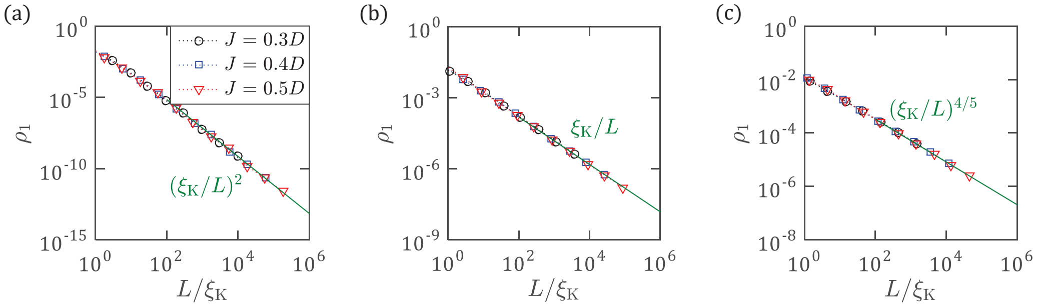

Supplementary Fig. 1. Universal scaling of the spatial distribution of Kondo clouds. Distribution ρ_1 is drawn as a function of L/ξ_K for various Kondo coupling J in (a) the single-channel Kondo (1CK), (b) the isotropic two-channel Kondo (2CK), and (c) the isotropic three-channel Kondo (3CK) effects. Data points from numerical renormalization (NRG) calculations with different values of J lie on a single curve well fitted by the boundary conformal field theory (BCFT) prediction of $\rho_1 \propto (\xi_K/L)^{2\Delta}$, showing the universal scaling of the cloud tail in the isotropic Kondo effects. The distribution on different channels is identical to ρ_1 in the isotropic Kondo effects. In the NRG calculation, we choose $J = 0.3D$, $B = 0.1D$, and the z-averaging of $z = 0$ and $1/2$.

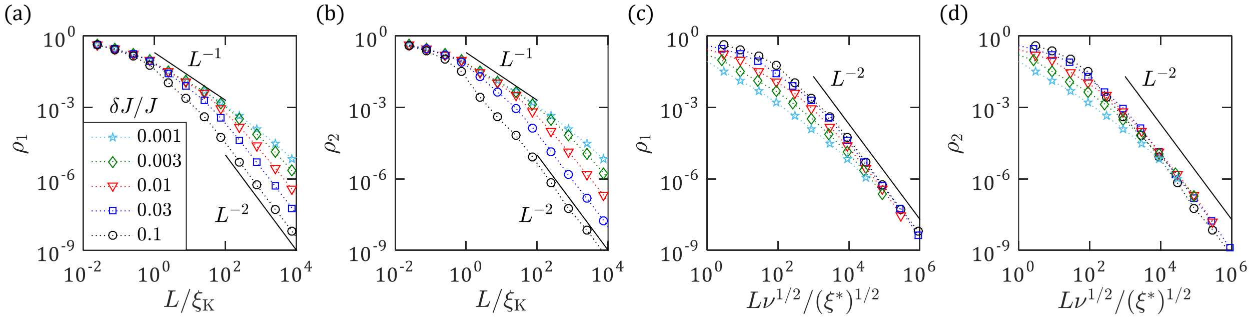

Supplementary Fig. 2. Universal scaling of the cloud distribution in the anisotropic two-channel Kondo (2CK) effect with $J_1 > J_2$. (a,b) Distribution ρ_1 and ρ_2 as a function of L/ξ_K for various channel anisotropy δJ. Crossover from the 2CK region of $\rho_{1,2} \propto L^{-1}$ to the single-channel Kondo (1CK) region of $\rho_1 \propto L^{-2}$ and the non-Kondo Fermi liquid of $\rho_2 \propto L^{-2}$ is shown. (c,d) The results are redrawn as a function of $L\nu^{1/2}/(\xi^*)^{1/2}$. The numerical renormalization group (NRG) results with various δJ lie on a single curve at $L > \xi^*$ in agreement with the bosonization prediction of $\rho_{1,2} \propto L^{-2}$. We choose $J_1 = J + \delta J$, $J_2 = J - \delta J$, $J = 0.4D$, $B = 0.1D$, and the z-averaging of $z = 0$ and $1/2$.

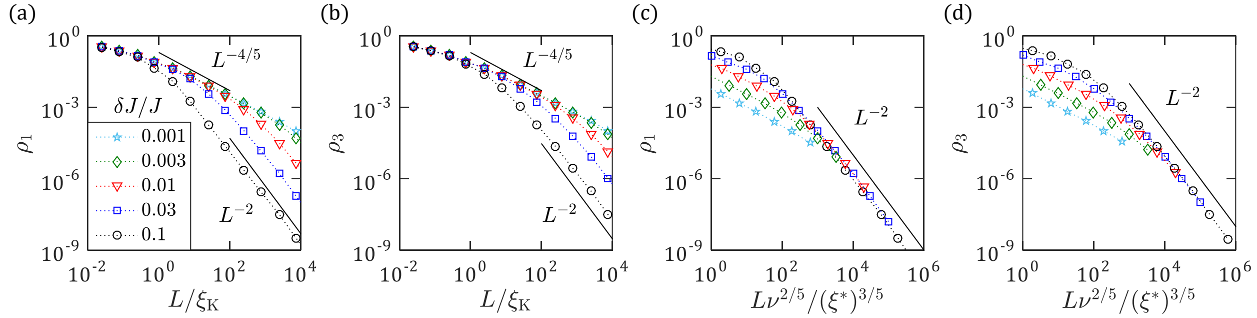

Supplementary Fig. 3. Universal scaling of the cloud distribution in the anisotropic three-channel Kondo (3CK) effect with $J_1 = J_2 < J_3$. (a,b) Distribution ρ_1 and ρ_3 as a function of L/ξ_{K} for various δJ. Crossover from the 3CK region of $\rho_{1,2,3} \propto L^{-4/5}$ to the single-channel Kondo (1CK) region of $\rho_3 \propto L^{-2}$ and the non-Kondo Fermi liquid of $\rho_{1,2} \propto L^{-2}$ is shown. (c,d) The results, redrawn as a function of $L\nu^{2/5}/(\xi^*)^{3/5}$, lie on a single curve $\propto L^{-2}$. We choose $J_{1,2} = J - \delta J/2$, $J_3 = J + \delta J$, $J = 0.4D$, $B = 0.1D$, and the z-averaging of $z = 0$ and $1/2$.

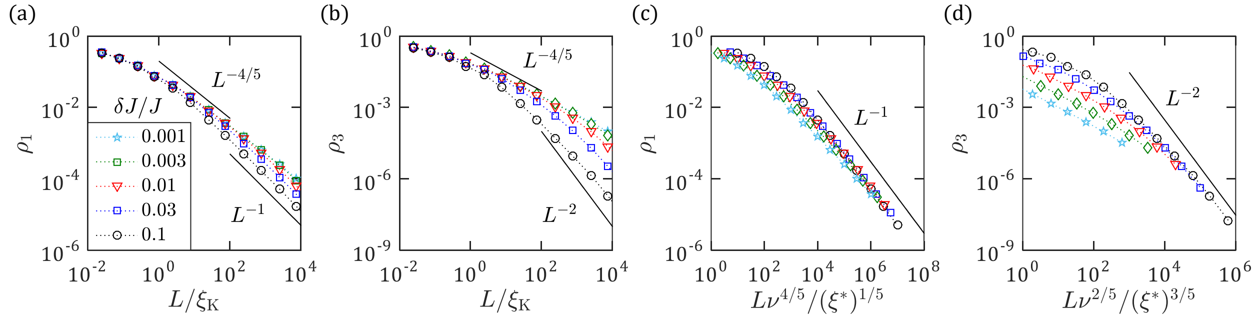

Supplementary Fig. 4. Universal scaling of the cloud distribution in the anisotropic three-channel Kondo (3CK) effect with $J_1 = J_2 > J_3$. (a,b) Distribution ρ_1 and ρ_3 as a function of L/ξ_{K} for various δJ. The distribution ρ_2 is equal to ρ_1. Crossover from the 3CK region of $\rho_{1,2,3} \propto L^{-4/5}$ to the two-channel Kondo (2CK) region of $\rho_{1,2} \propto L^{-1}$ and the non-Kondo Fermi liquid of $\rho_3 \propto L^{-2}$ is shown. (c,d) The results are redrawn as a function of $L\nu^{4/5}/(\xi^*)^{1/5}$ for ρ_1 and $L\nu^{2/5}/(\xi^*)^{3/5}$ for ρ_3. They lie on a single curve $\propto L^{-1}$ for ρ_1 and $\propto L^{-2}$ for ρ_3. We choose $J_{1,2} = J + \delta J/2$, $J_3 = J - \delta J$, $J = 0.4D$, $B = 0.1D$, and the z-averaging of $z = 0$ and $1/2$.

Supplementary Note 4. ENTANGLEMENT SHELLS OF KONDO CLOUD WITH GENERAL CHANNEL ANISOTROPY

We present the spatial distribution of Kondo clouds in the 3CK effects where the coupling strengths all are different, $J_1 > J_2 > J_3$. The shell structure of the distribution is found in Supplementary Fig. 5. The result shows that the shell structure reflects crossover between different Kondo fixed points. From the innermost region, there occur four different shells. All the channels have the core region in common, which corresponds to the local moment phase. The core region is followed by the 3CK shell in all the channels. The next outer shell depends on the relative coupling strengths. When $J_1 > J_2 > J_3$ or $J_1 = J_2 > J_3$, the channels 1 and 2 have the 2CK region in the shell, while the channel 3 has the non-Kondo Fermi liquid region. When $J_1 > J_2 = J_3$, the channel 1 has the 1CK region in the shell, while the channels 2 and 3 have the non-Kondo Fermi liquid region. This shell is the outermost shell when $J_1 = J_2 > J_3$ or $J_1 > J_2 = J_3$. When $J_1 > J_2 > J_3$, there occurs another shell in the outmost region, which shows the 1CK region in the channel 1 and the non-Kondo Fermi liquid region in the channels 2 and 3.

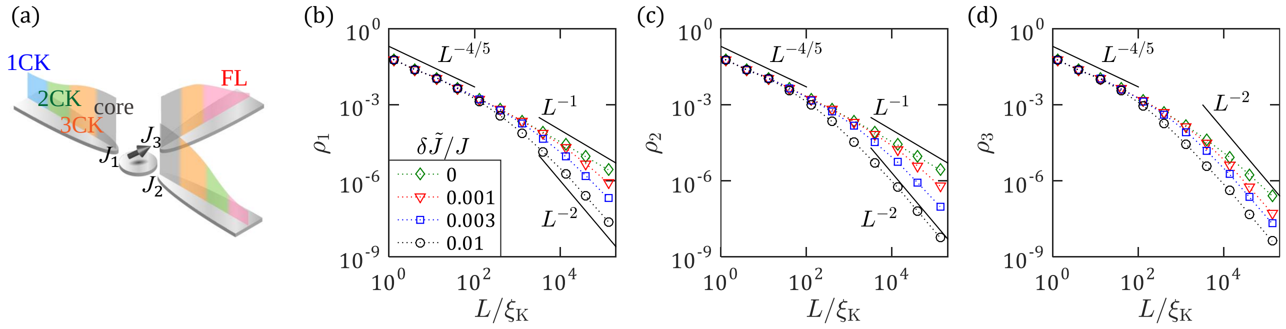

Supplementary Fig. 5. Shell structure of Kondo clouds in the anisotropic three-channel Kondo (3CK) effect with $J_1 > J_2 > J_3$. (a) Schematic view of the cloud distribution. (b-d) Distribution ρ_1, ρ_2, and ρ_3 for different channel anisotropy $\delta\tilde{J}$. Different shells are identified by using the power law exponents of the dependence of the cloud distribution on L. We choose $J_1 = J(1+\delta J/2J)(1+\delta\tilde{J}/J)$, $J_2 = J(1+\delta J/2J)(1-\delta\tilde{J}/J)$, $J_3 = J - \delta J$ where $J = 0.3D$, $\delta J = 0.01J$, $B = 0.1D$, and the z-averaging of $z = 0$ and $1/2$.

The result of the channel-anisotropic 3CK effect is well generalized to the shell structure of the Kondo cloud distribution in the kCK effects with $k \geq 4$. The core region is followed by the next inner kCK shell. Then there occur k''CK, q_1CK, q_2CK, $\cdots$, kCK shells from the outermost to innermost shells, where $1 \leq k'' < q_1 < q_2 < \cdots < k$. The values of $k'' < q_1 < q_2 < \cdots < k$ are determined by the relative Kondo coupling strengths. In each intermediate shell, e.g., in the qCK shell, the channels $1, 2, \cdots, q$ of stronger coupling strengths show qCK region, while the other channels of weaker coupling exhibit the non-Kondo Fermi liquid region.

Supplementary Note 5. DERIVATION OF EQ. (5)

We derive Eq. (5) of the main text. To do this, we first derive the magnetization $\langle \mathbf{S}_{\mathrm{imp}} \rangle$ of the impurity in the presence of the local spin symmetry breaking H_{LSB}. The kCK Hamiltonian $H_{k\mathrm{CK}} = \sum_{j=1}^{k} J_j \mathbf{S}_{\mathrm{imp}} \cdot \mathbf{S}_j(0) + H_j$ with isotropic couplings $J_1 = \cdots = J_k = J$ at $T \ll T_K$ is described by the boundary conformal field theory (BCFT) Hamiltonian

$$H_{\mathrm{BCFT}} = H_{\mathrm{FP}} + \lambda H_{\mathrm{LI}}. \tag{S13}$$

H_{FP} is the fixed-point Hamiltonian invariant under $\mathrm{U}(1) \times \mathrm{SU}(2)_k \times \mathrm{SU}(k)_2$ Kac-Moody algebra. λH_{LI} is the leading irrelevant operator with coupling constant $\lambda \propto 1/T_{\mathrm{K}}^{\Delta}$. According to the Kac-Moody symmetry, operators are labeled by the representation (quantum numbers of charge Q, spin J_s, flavor J_f) of three sectors $\mathrm{U}(1)$, $\mathrm{SU}(2)_k$, $\mathrm{SU}(k)_2$. The identity operator I corresponds to the trivial representation. The spin (resp. flavor)-adjoint primary boundary operator $\boldsymbol{\phi}_s$ (resp. $\boldsymbol{\phi}_f$) corresponds to adjoint representation of $\mathrm{SU}(2)_k$ (resp. $\mathrm{SU}(k)_2$) with conformal dimension $\Delta = 2/(k+2)$ (resp. $1 - \Delta$). The local spin symmetry breaking perturbation H_{LSB} in the n-th channel, shown in Eq. (S10), has the equivalent form of

$$H_{\mathrm{LSB}} = \frac{B}{2}\left(\psi_{\uparrow n}^{\dagger}(L)\psi_{\downarrow n}(L) + \psi_{\downarrow n}^{\dagger}(L)\psi_{\uparrow n}(L)\right). \tag{S14}$$

Here $\alpha = \uparrow, \downarrow$ represents the eigenspin states of the spin operator S^z in the z direction. The perturbation acts like a local magnetic field at position $x = L$ in the x-direction as shown in Eq. (S10) so that the magnetization of the impurity has only the x-direction component $\langle S_{\mathrm{imp}}^x \rangle$; namely

$$\langle S_{\mathrm{imp}}^{y,z} \rangle = 0. \tag{S15}$$

Below we derive $\langle S_{\mathrm{imp}}^x \rangle$, based on BCFT. The result is in good agreement with the NRG calculation in Supplementary Fig. 6.

In the derivation, we use a chiral field. We decompose the field $\psi_{\alpha j}(x)$ annihilating an electron with spin α at channel j into the left and right moving chiral fields, $\psi_{\mathrm{L}\alpha j}(x)$ and $\psi_{\mathrm{R}\alpha j}(x)$,

$$\psi_{\alpha j}(x) = e^{-ik_F x}\psi_{\mathrm{L}\alpha j}(x) + e^{ik_F x}\psi_{\mathrm{R}\alpha j}(x) \qquad \alpha = \uparrow, \downarrow, \quad j = 1, \cdots, k, \tag{S16}$$

for $x \geq 0$. By defining $\psi_{\mathrm{L}\alpha j}(x) = \psi_{\mathrm{R}\alpha j}(-x)$ for $x < 0$, $\psi_{\alpha j}(x)$ is expressed in terms of one chiral field as $\psi_{\alpha j}(x) = e^{-ik_F x}\psi_{\mathrm{L}\alpha j}(x) + e^{ik_F x}\psi_{\mathrm{L}\alpha j}(-x)$.

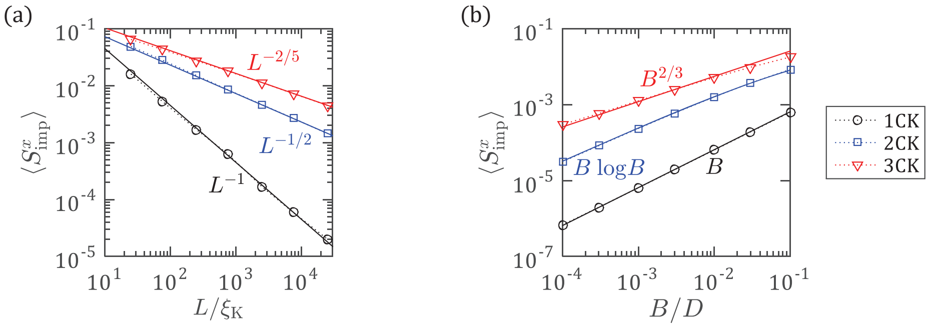

Supplementary Fig. 6. The expectation value $\langle S_{\mathrm{imp}}^x \rangle$ of the impurity spin in the x direction at zero temperature, in the single-channel Kondo (1CK) (black), the isotropic two-channel Kondo (2CK) (blue), and the isotropic three-channel Kondo (3CK) (red) effect in the presence of the spin symmetry breaking perturbation at position L in the $n = 1$ channel. Its dependence on the perturbation position L and strength B is shown in (a) and (b). Data points, obtained by the numerical renormalization group (NRG) calculation, agree with the fitting curves predicted by the boundary conformal field theory (BCFT). In the NRG, we use $J = 0.3D$, $B = 0.1D$ [for computing the result in (a)], and the z-averaging of $z = 0$ and $1/2$.

A. Multichannel Kondo Model

We calculate $\langle S^x_{\rm imp}\rangle$ in the case of $k \geq 2$. In this case, the leading irrelevant operator $H_{\rm LI} = \mathbf{J}_{-1}\cdot\boldsymbol{\phi}_s$ is the Kac-Moody descendant of the spin adjoint primary boundary operator $\boldsymbol{\phi}_s$, and non-perturbative treatment is required in computing $\langle S^x_{\rm imp}\rangle$ at zero temperature. In the Lagrangian description, the action corresponding to the Hamiltonian $H_{\rm FP} + \lambda H_{\rm LI} + H_{\rm LSB}$ is written as

$$\begin{aligned} S = S_{\rm FP} &+ \lambda \int_{-\infty}^{\infty} d\tau\, \mathbf{J}_{-1}\cdot\boldsymbol{\phi}_s(\tau,0) \\ &+ \frac{B}{2}\int_{-\infty}^{\infty} d\tau \Bigg(J^x_{{\rm L}n}(\tau,L) + J^x_{{\rm L}n}(\tau,-L) + e^{2ik_FL}\Big(\psi^\dagger_{{\rm L}\uparrow n}(\tau,L)\psi_{{\rm L}\downarrow n}(\tau,-L) + \psi^\dagger_{{\rm L}\downarrow n}(\tau,L)\psi_{{\rm L}\uparrow n}(\tau,-L)\Big) \\ &\qquad + e^{-2ik_FL}\Big(\psi^\dagger_{{\rm L}\uparrow n}(\tau,-L)\psi_{{\rm L}\downarrow n}(\tau,L) + \psi^\dagger_{{\rm L}\downarrow n}(\tau,-L)\psi_{{\rm L}\uparrow n}(\tau,L)\Big)\Bigg). \end{aligned} \tag{S17}$$

$S_{\rm FP}$ is the action at the fixed point corresponding to $H_{\rm FP}$. τ is the Euclidean time. $J^x_{{\rm L}n}(\tau,x) \equiv \psi^\dagger_{{\rm L}\uparrow n}(\tau,x)\psi_{{\rm L}\downarrow n}(\tau,x) + \psi^\dagger_{{\rm L}\downarrow n}(\tau,x)\psi_{{\rm L}\uparrow n}(\tau,x)$ is the x-component of the local spin. The terms proportional to B come from $H_{\rm LSB}$, and they are written in terms of the chiral field $\psi_{\alpha j}(x) = e^{-ik_Fx}\psi_{{\rm L}\alpha j}(x) + e^{ik_Fx}\psi_{{\rm L}\alpha j}(-x)$ defined below Eq. (S16). We obtain $\langle S^x_{\rm imp}\rangle$ by using the series expansion in the perturbation theory,

$$\langle S^x_{\rm imp}\rangle = \frac{1}{\mathcal{Z}}\sum_{m_1,m_2,m_3=0}^{\infty}\frac{1}{m_1!m_2!m_3!}A_{m_1,m_2,m_3}, \tag{S18}$$

$$\begin{aligned} A_{m_1,m_2,m_3} = \Bigg\langle S^x_{\rm imp}\Big(-\lambda\int_{-\infty}^{\infty} d\tau\,\mathbf{J}_{-1}\cdot\boldsymbol{\phi}_s(\tau,0)\Big)^{m_1}\Big(-\frac{B}{2}\int_{-\infty}^{\infty} d\tau\Big(J^x_{{\rm L}n}(\tau,L)+J^x_{{\rm L}n}(\tau,-L)\Big)\Big)^{m_2} \\ \times\Bigg(-\frac{B}{2}\int_{-\infty}^{\infty} d\tau\Big[e^{2ik_FL}\Big(\psi^\dagger_{{\rm L}\uparrow n}(\tau,L)\psi_{{\rm L}\downarrow n}(\tau,-L) + \psi^\dagger_{{\rm L}\downarrow n}(\tau,L)\psi_{{\rm L}\uparrow n}(\tau,-L)\Big) \\ + e^{-2ik_FL}\Big(\psi^\dagger_{{\rm L}\uparrow n}(\tau,-L)\psi_{{\rm L}\downarrow n}(\tau,L) + \psi^\dagger_{{\rm L}\downarrow n}(\tau,-L)\psi_{{\rm L}\uparrow n}(\tau,L)\Big)\Big]\Bigg)^{m_3}\Bigg\rangle_{\rm FP}. \end{aligned} \tag{S19}$$

$\langle\cdots\rangle_{\rm FP}$ is the expectation value with respect to the fixed point action $S_{\rm FP}$. $\mathcal{Z}$ is the partition function of the action S in Eq. (S17). The impurity spin is identified with the spin adjoint primary operator ϕ^x_s,

$$S^x_{\rm imp} = \frac{s_1}{T_{\rm K}^{\Delta}}\phi^x_s + \sum_i \psi_i, \tag{S20}$$

where s_1 is a constant and ψ_i's indicate the scaling fields of dimension $> \Delta$. In the expression of A_{m_1,m_2,m_3}, we rescale the time and space by $(\tau,x) \to (\tau',x') = (a/L)(\tau,x)$ with $a/L \ll 1$. Using the covariance of the fields under the scale transformation, A_{m_1,m_2,m_3} becomes

$$\begin{aligned} A_{m_1,m_2,m_3} = \frac{1}{(L/a)^{\Delta+m_1\Delta}}\Bigg\langle S^x_{\rm imp}\Big(-\lambda\int_{-\infty}^{\infty} d\tau'\,\mathbf{J}_{-1}\cdot\boldsymbol{\phi}_s(\tau',0)\Big)^{m_1}\Big(-\frac{B}{2}\int_{-\infty}^{\infty} d\tau'\Big(J^x_{{\rm L}n}(\tau',a)+J^x_{{\rm L}n}(\tau',-a)\Big)\Big)^{m_2} \\ \times\Bigg(-\frac{B}{2}\int_{-\infty}^{\infty} d\tau\Big[e^{2ik_FL}\Big(\psi^\dagger_{{\rm L}\uparrow n}(\tau',a)\psi_{{\rm L}\downarrow n}(\tau',-a) + \psi^\dagger_{{\rm L}\downarrow n}(\tau',a)\psi_{{\rm L}\uparrow n}(\tau',-a)\Big) \\ + e^{-2ik_FL}\Big(\psi^\dagger_{{\rm L}\uparrow n}(\tau',-a)\psi_{{\rm L}\downarrow n}(\tau',a) + \psi^\dagger_{{\rm L}\downarrow n}(\tau,-a)\psi_{{\rm L}\uparrow n}(\tau',a)\Big)\Big]\Bigg)^{m_3}\Bigg\rangle_{\rm FP}. \end{aligned} \tag{S21}$$

Here we utilized the fact that the scaling dimensions of ϕ^x_s and $\mathbf{J}_{-1}\cdot\boldsymbol{\phi}_s$ are $\Delta = 2/(k+2)$ and $1+\Delta$, respectively. In the BCFT description of the multichannel Kondo model [S9], we have the operator product expansion (OPE)

$$\psi^\dagger_{{\rm L}\uparrow n}(\tau',a)\psi_{{\rm L}\downarrow n}(\tau',-a) + \psi^\dagger_{{\rm L}\downarrow n}(\tau',a)\psi_{{\rm L}\uparrow n}(\tau',-a) \to \frac{c_1}{a^{1-\Delta}}\phi^x_s(\tau',0) + \cdots \tag{S22}$$

where c_1 is a constant and $\cdots$ represents the scaling fields with dimension larger than $\Delta = 2/(k+2)$. Applying this to Eq. (S21), we obtain

$$A_{m_1,m_2,m_3} = \frac{1}{(L/a)^{\Delta+m_1\Delta}}\Bigg\langle S^x_{\rm imp}\Bigg(-\lambda\int_{-\infty}^{\infty}d\tau'\,\mathbf{J}_{-1}\cdot\boldsymbol{\phi}_s(\tau',0)\Bigg)^{m_1}\Bigg(-\frac{B}{2}\int_{-\infty}^{\infty}d\tau'\Big(J^x_{{\rm L}n}(\tau',a)+J^x_{{\rm L}n}(\tau',-a)\Big)\Bigg)^{m_2} \times\Bigg(-\frac{B}{2}\int_{-\infty}^{\infty}d\tau\Big[e^{2ik_FL}\Big(\frac{c_1}{a^{1-\Delta}}\phi^x_s(\tau',0)\Big)+e^{-2ik_FL}\Big(\frac{c_1}{a^{1-\Delta}}\phi^x_s(\tau',0)\Big)\Big]\Bigg)^{m_3}\Bigg\rangle_{\rm FP} \tag{S23}$$

We again rescale the time and space by $(\tau',x')\to(\tau,x)=(L/a)(\tau',x')$ and return to the original ones.

$$A_{m_1,m_2,m_3} = \Bigg\langle S^x_{\rm imp}\Bigg(-\lambda\int_{-\infty}^{\infty}d\tau\,\mathbf{J}_{-1}\cdot\boldsymbol{\phi}_s(\tau,0)\Bigg)^{m_1}\Bigg(-\frac{B}{2}\int_{-\infty}^{\infty}d\tau\Big(J^x_{{\rm L}n}(\tau,L)+J^x_{{\rm L}n}(\tau,-L)\Big)\Bigg)^{m_2} \times\Bigg(-\frac{c_1B}{L^{1-\Delta}}\cos(2k_FL)\int_{-\infty}^{\infty}d\tau\,\phi^x_s(\tau,0)\Bigg)^{m_3}\Bigg\rangle_{\rm FP}. \tag{S24}$$

This means that the expectation value is perturbed by the leading irrelevant operator $\lambda\mathbf{J}_{-1}\cdot\boldsymbol{\phi}_s$, the magnetic field $B(J^x_{{\rm L}n}(L)+J^x_{{\rm L}n}(-L))$, and $(c_1B/L^{1-\Delta})\cos(2k_FL)\phi^x_s(0)$. Since $\phi^x_s(\tau',0)$ has smaller scaling dimension than $J^x_{{\rm L}n}(\tau',\pm a)$, the contribution from $\phi^x_s(\tau',0)$ dominates over that from $J^x_{{\rm L}n}(\tau',\pm a)$. Using Eq. (S20), we have

$$\frac{c_1B}{L^{1-\Delta}}\cos(2k_FL)\phi^x_s(0)\simeq\frac{c_1B}{L^{1-\Delta}}\cos(2k_FL)\frac{T_{\rm K}^{\Delta}}{s_1}S^x_{\rm imp}\equiv B^xS^x_{\rm imp}. \tag{S25}$$

This shows that the local spin symmetry breaking perturbation $H_{\rm LSB}$ is equivalent, in the calculation of $\langle S^x_{\rm imp}\rangle$, with the perturbation by the x-directional magnetic field B^x applied to the magnetic impurity at low temperature near the fixed point. It is known [S10, S11] that the magnetic field results in

$$\langle S^x_{\rm imp}\rangle\propto\begin{cases}B^x\log B^x & k=2\\ (B^x)^{2/k} & k\ge 3\end{cases} \tag{S26}$$

at zero temperature. Thus, Eq. (S25) and Eq. (S26) gives the leading order of $\langle S^x_{\rm imp}\rangle$:

$$\langle S^x_{\rm imp}\rangle\propto\begin{cases}(B\log B)(\xi_{\rm K}/L)^{1/2}\cos(2k_FL) & k=2\\ B^{2/k}(\xi_{\rm K}/L)^{2/(k+2)}(\cos(2k_FL))^{2/k} & k\ge 3\end{cases} \tag{S27}$$

where $\xi_{\rm K}=1/T_{\rm K}$.

The LSB breaks the SU(2) spin symmetry, making the ground state non-degenerate. So we can use Eq. (3) of the main text, since it is applicable for a pure state such as the non-degenerate ground state. By using Eq. (S15), we have

$$\mathcal{N}(L,T=0;n)=\sqrt{1-\frac{4\mathbf{M}^2}{\hbar^2}}=\sqrt{1-\frac{4\langle S^x_{\rm imp}\rangle^2}{\hbar^2}}\simeq 1-\frac{2\langle S^x_{\rm imp}\rangle^2}{\hbar^2},\qquad L\gg\xi_{\rm K}. \tag{S28}$$

By using Eq. (2) of the main text and Eqs. (S27) and (S28), we obtain

$$\rho_n(L,T=0)\equiv\mathcal{N}_0(T=0)-\mathcal{N}(L,T=0;n)\simeq\frac{2\langle S^x_{\rm imp}\rangle^2}{\hbar^2}\propto(\frac{\xi_{\rm K}}{L})^{\frac{4}{k+2}}(\cos(2k_FL))^{4/k},\qquad L\gg\xi_{\rm K}. \tag{S29}$$

Here we use $\mathcal{N}_0(T=0)=1$ in the absence of the LSB. We focus on the envelop of the L dependence in Eq. (S29), because it exhibits a universality of the Kondo cloud:

$$\rho_n(L,T=0)\propto(\frac{\xi_{\rm K}}{L})^{\frac{4}{k+2}},\qquad L\gg\xi_{\rm K}. \tag{S30}$$

It is Eq. (5) for $k\ge 2$ in the main text. Here we choose the value $2k_FL$ as an integer multiple of π, which give the maximum value $|\cos(2k_FL)|=1$, to trace the envelope and clearly show the power decay of the Kondo cloud.

B. Single Channel Kondo Model

We calculate $\langle S^x_{\rm imp}\rangle$ in the single channel Kondo model. Here, the leading irrelevant operator is given by the local spin product $H_{\rm LI} = \mathbf{J}_{\rm L}\cdot\mathbf{J}_{\rm L}(0)$. Similar to Eq. (S17), we have the action corresponding to the Hamiltonian $H_{\rm FP} + \lambda H_{\rm LI} + H_{\rm LSB}$ as

$$\begin{aligned} S = S_{\rm FP} &+ \lambda \int_{-\infty}^{\infty} d\tau\, \mathbf{J}_{\rm L}\cdot\mathbf{J}_{\rm L}(\tau,0) \\ &+ \frac{B}{2}\int_{-\infty}^{\infty} d\tau \Bigg(J^x_{\rm L}(\tau,L) + J^x_{\rm L}(\tau,-L) + e^{2ik_F L}\Big(\psi^\dagger_{\rm L\uparrow}(\tau,L)\psi_{\rm L\downarrow}(\tau,-L) + \psi^\dagger_{\rm L\downarrow}(\tau,L)\psi_{\rm L\uparrow}(\tau,-L)\Big) \\ &\qquad + e^{-2ik_F L}\Big(\psi^\dagger_{\rm L\uparrow}(\tau,-L)\psi_{\rm L\downarrow}(\tau,L) + \psi^\dagger_{\rm L\downarrow}(\tau,-L)\psi_{\rm L\uparrow}(\tau,L)\Big)\Bigg) \end{aligned} \tag{S31}$$

Here we omit the channel index n because there is only one channel. The impurity spin is identified with the spin density operator of scaling dimension 1,

$$S^x_{\rm imp} = \frac{s_2}{T_{\rm K}} J^x_{\rm L} + \sum_i \psi_i, \tag{S32}$$

where s_2 is a constant and ψ_i's indicate the scaling fields of dimension > 1. In this 1CK case, perturbative treatment with respect to the terms proportional to B is possible in computing $\langle S^x_{\rm imp}\rangle$ at zero temperature, contrary to the kCK case with $k \geq 2$. The leading contribution in the perturbation expansion of $\langle S^x_{\rm imp}\rangle$ with respect to the action terms proportional to B is given by

$$\begin{aligned} \langle S^x_{\rm imp}\rangle &\simeq -\int_{-\infty}^{\infty} d\tau \Bigg\langle S^x_{\rm imp}\frac{B}{2}\Bigg(e^{2ik_F L}\Big(\psi^\dagger_{\rm L\uparrow}(\tau,L)\psi_{\rm L\downarrow}(\tau,-L) + \psi^\dagger_{\rm L\downarrow}(\tau,L)\psi_{\rm L\uparrow}(\tau,-L)\Big) \\ &\qquad + e^{-2ik_F L}\Big(\psi^\dagger_{\rm L\uparrow}(\tau,-L)\psi_{\rm L\downarrow}(\tau,L) + \psi^\dagger_{\rm L\downarrow}(\tau,-L)\psi_{\rm L\uparrow}(\tau,L)\Big)\Bigg)\Bigg\rangle \\ &\simeq -\int_{-\infty}^{\infty} d\tau \Bigg\langle \frac{s_2}{T_{\rm K}} J^x_{\rm L}(0,0)\frac{B}{2}\Bigg(e^{2ik_F L}\Big(\psi^\dagger_{\rm L\uparrow}(\tau,L)\psi_{\rm L\downarrow}(\tau,-L) + \psi^\dagger_{\rm L\downarrow}(\tau,L)\psi_{\rm L\uparrow}(\tau,-L)\Big) \\ &\qquad + e^{-2ik_F L}\Big(\psi^\dagger_{\rm L\uparrow}(\tau,-L)\psi_{\rm L\downarrow}(\tau,L) + \psi^\dagger_{\rm L\downarrow}(\tau,-L)\psi_{\rm L\uparrow}(\tau,L)\Big)\Bigg)\Bigg\rangle \\ &\propto \frac{\pi s_2}{T_K L} B\cos(2k_F L). \end{aligned} \tag{S33}$$

In the last equality, we used the three-point correlator $\langle J^x_{\rm L}\psi^\dagger\psi\rangle$ in BCFT. Hence, $\langle S^x_{\rm imp}\rangle \sim O(B(\xi_{\rm K}/L))$ in the 1CK.

By following the way to derive Eqs. (S28) and (S29), we obtain

$$\rho_n(L, T=0) \equiv \mathcal{N}_0(T=0) - \mathcal{N}(L, T=0; n) \simeq \frac{2\langle S^x_{\rm imp}\rangle^2}{\hbar^2} \propto (\frac{\xi_{\rm K}}{L})^2\cos^2(2k_F L), \qquad L \gg \xi_{\rm K}. \tag{S34}$$

We focus on the envelope of the Friedel oscillation as in Eqs. (S30) and (S29):

$$\rho_n(L, T=0) \propto (\frac{\xi_{\rm K}}{L})^2, \qquad L \gg \xi_{\rm K}. \tag{S35}$$

It is Eq. (5) for the single channel Kondo model in the main text.

Supplementary Note 6. CHANNEL ANISOTROPIC 2CK EFFECTS

We compute $\langle S^x_{\rm imp}\rangle$ in the channel anisotropic 2CK effect in the presence of the local symmetry breaking perturbation. The total Hamiltonian is $H + \sum_{n=1,2} H_{{\rm LSB},n}$. Here H describes the channel anisotropic 2CK effect,

$$H = H_1 + H_2 + \sum_{j=1,2} J S^z_{\rm imp} S^z_i(0) + \sum_{j=1,2}\sum_{\alpha=x,y} (J + (-1)^i \delta J) S^\alpha_{\rm imp} S^\alpha_j(0), \tag{S36}$$

where $H_{1(2)}$ is the Hamiltonian for free electrons in the channel 1 (2). $H_{\mathrm{LSB},n}$ describes the local symmetry breaking perturbation on channel $n = 1, 2$ in Eq. (S14). Using the chiral field in Eq. (S16), we rewrite $H_{\mathrm{LSB},n}$,

$$H_{\mathrm{LSB},n} = \frac{B}{2}\Bigg(J^x_{\mathrm{L}n}(L) + J^x_{\mathrm{L}n}(-L) + e^{2ik_F L}\Big(\psi^\dagger_{\mathrm{L}\uparrow n}(L)\psi_{\mathrm{L}\downarrow n}(-L) + \psi^\dagger_{\mathrm{L}\downarrow n}(L)\psi_{\mathrm{L}\uparrow n}(-L)\Big) + e^{-2ik_F L}\Big(\psi^\dagger_{\mathrm{L}\uparrow n}(-L)\psi_{\mathrm{L}\downarrow n}(L) + \psi^\dagger_{\mathrm{L}\downarrow n}(-L)\psi_{\mathrm{L}\uparrow n}(L)\Big)\Bigg). \tag{S37}$$

We use the bosonization method [S12, S13]. The fermion field is bosonized, $\psi_{\mathrm{L}\alpha j}(x) \propto e^{-i\varphi_{\alpha j}(x)}$. The fields $\varphi_c = \frac{1}{2}(\varphi_{\uparrow 1} + \varphi_{\downarrow 1} + \varphi_{\uparrow 2} + \varphi_{\downarrow 2})$, $\varphi_s = \frac{1}{2}(\varphi_{\uparrow 1} - \varphi_{\downarrow 1} + \varphi_{\uparrow 2} - \varphi_{\downarrow 2})$, $\varphi_f = \frac{1}{2}(\varphi_{\uparrow 1} + \varphi_{\downarrow 1} - \varphi_{\uparrow 2} - \varphi_{\downarrow 2})$, $\varphi_{sf} = \frac{1}{2}(\varphi_{\uparrow 1} - \varphi_{\downarrow 1} - \varphi_{\uparrow 2} + \varphi_{\downarrow 2})$ in charge, spin, flavor, spin-flavor sectors are introduced. Emery-Kivelson transformation $U = e^{iS^z_{\mathrm{imp}}\varphi_s(0)}$ is applied to H, $\widetilde{H} = UHU^\dagger$. Refermionization decomposes $\widetilde{H}$ into three free fermion Hamiltonians and one resonant level model,

$$UHU^\dagger = \sum_{A=c,s,f,sf} \int dx\, \psi^\dagger_A(x) i\partial\psi_A(x) + \sqrt{\Gamma}(\psi_{sf}(0) + \psi^\dagger_{sf}(0))(c_d - c^\dagger_d) + \sqrt{\delta\Gamma}(\psi_{sf}(0) - \psi^\dagger_{sf}(0))(c_d + c^\dagger_d) \tag{S38}$$

where c_d is a local pseudofermion describing the impurity spin through $S^z_{\mathrm{imp}} = c^\dagger_d c_d - 1/2$ and $S^-_{\mathrm{imp}} = S^x_{\mathrm{imp}} - iS^y_{\mathrm{imp}} = F_s c_d$ with the Klein factor F_s of the spin sector, $\psi_A(x) \propto e^{-\varphi_A(x)}$ is a fermion field in the $A = c, s, f, sf$ sector, $\Gamma = J^2/4a$, $\delta\Gamma = (\delta J)^2/4a$, and a is a short distance cutoff. The impurity spin operator and the fermion field are decomposed into Majorana fermions $\hat{a}$, $\hat{b}$, χ_A, η_A as

$$\hat{a} = \frac{c_d + c^\dagger_d}{\sqrt{2}}, \qquad \hat{b} = \frac{c_d - c^\dagger_d}{\sqrt{2}i}, \qquad \chi_A(x) = \frac{\psi_A(x) + \psi^\dagger_A(x)}{\sqrt{2}}, \qquad \eta_A(x) = \frac{\psi_A(x) - \psi^\dagger_A(x)}{\sqrt{2}i}, \qquad A = c, s, f, sf. \tag{S39}$$

The Hamiltonian is rewritten in terms of the Majorana fermions as

$$UHU^\dagger = \sum_{A=c,s,f,sf}\left(\frac{1}{2}\int dx\, \chi^\dagger_A(x) i\partial\chi_A(x) + \frac{1}{2}\int dx\, \eta^\dagger_A(x) i\partial\eta_A(x)\right) + 2i\sqrt{\Gamma}\chi_{sf}(0)\hat{b} + 2i\sqrt{\delta\Gamma}\eta_{sf}(0)\hat{a}. \tag{S40}$$

The quadratic Hamiltonian for the spin-flavor sector is diagonalized as

$$\frac{1}{2}\int dx\, \widetilde{\chi}^\dagger_{sf}(x) i\partial\widetilde{\chi}_{sf}(x) + \frac{1}{2}\int dx\, \widetilde{\eta}^\dagger_{sf}(x) i\partial\widetilde{\eta}_{sf}(x), \tag{S41}$$

where $\widetilde{\chi}_{sf}$ is related with χ_{sf} and $\hat{b}$ and $\widetilde{\eta}_{sf}$ is related with η_{sf} and $\hat{a}$. In the limit of low energy ($\ll T_{\mathrm{K}}$) or $\Gamma, \delta\Gamma \to \infty$, the modified Majorana fermions $\widetilde{\chi}_{sf}$ and $\widetilde{\eta}_{sf}$ absorb the Majorana fermions $\hat{b}$ and $\hat{a}$ of the impurity, respectively, satisfying $\widetilde{\chi}_{sf}(x) = \chi_{sf}(x)\mathrm{sgn}(x)$ and $\widetilde{\eta}_{sf}(x) = \eta_{sf}(x)\mathrm{sgn}(x)$ at $x \neq 0$ and

$$\widetilde{\chi}_{sf}(0) = \sqrt{\Gamma}\hat{b}, \qquad \widetilde{\eta}_{sf}(0) = \sqrt{\delta\Gamma}\hat{a}. \tag{S42}$$

Hence the modified Majorana fermions obey a modified boundary condition [S14]. After the absorption, the boson field $\widetilde{\varphi}_{sf}(x)$ corresponding to the modified fermion field $\widetilde{\psi}_{sf}(x) \equiv \widetilde{\chi}_{sf}(x) + i\widetilde{\eta}_{sf}(x) \sim F_{sf}e^{-i\widetilde{\varphi}_{sf}(x)}$ satisfies [S15, S16]

$$\widetilde{\varphi}_{sf}(0^+) = \widetilde{\varphi}_{sf}(0^-) + \pi. \tag{S43}$$

Furthermore, the Emery-Kivelson transformation U is a boundary condition changing operator, and the boson field φ_s in the spin sector is affected by U. The transformed boson field $\widetilde{\varphi}_s$ in the spin sector satisfies [S15, S16]

$$\widetilde{\varphi}_s(0^+) = \widetilde{\varphi}_s(0^-) + \pi. \tag{S44}$$

Therefore, the Hamiltonian H is equivalent with the free theory described by the boson fields φ_c, $\widetilde{\varphi}_s$, φ_f and $\widetilde{\varphi}_{sf}$ with the modified boundary conditions in Eq. (S43) and Eq. (S44).

According to Eqs. (S39) and (S42), the x-component of the impurity spin operator is expressed as

$$\begin{aligned}\widetilde{S}^x_{\mathrm{imp}} \equiv US^x_{\mathrm{imp}}U^{-1} &= e^{i\varphi_s(0)}\frac{\hat{a} - i\hat{b}}{2\sqrt{2}}F^\dagger_s + F_s\frac{\hat{a} + i\hat{b}}{2\sqrt{2}}e^{-i\varphi_s(0)} \\ &= -\frac{1}{4\sqrt{a}}\left(\frac{1}{\sqrt{\delta\Gamma}} + \frac{1}{\sqrt{\Gamma}}\right)F_{sf}F^\dagger_s e^{i\widetilde{\varphi}_s(0)}e^{-i\widetilde{\varphi}_{sf}(0)} + \frac{1}{4\sqrt{a}}\left(\frac{1}{\sqrt{\delta\Gamma}} - \frac{1}{\sqrt{\Gamma}}\right)F^\dagger_{sf}F^\dagger_s e^{i\widetilde{\varphi}_s(0)}e^{i\widetilde{\varphi}_{sf}(0)} \\ &\quad + \frac{1}{4\sqrt{a}}\left(\frac{1}{\sqrt{\delta\Gamma}} - \frac{1}{\sqrt{\Gamma}}\right)F_sF_{sf}e^{-i\widetilde{\varphi}_{sf}(0)}e^{-i\widetilde{\varphi}_s(0)} - \frac{1}{4\sqrt{a}}\left(\frac{1}{\sqrt{\delta\Gamma}} + \frac{1}{\sqrt{\Gamma}}\right)F_sF^\dagger_{sf}e^{i\widetilde{\varphi}_{sf}(0)}e^{-i\widetilde{\varphi}_s(0)}.\end{aligned} \tag{S45}$$

The local spin symmetry breaking perturbation in channel n is Emery-Kivelson transformed, $\widetilde{H}_{\mathrm{LSB},n} = UH_{\mathrm{LSB},n}U^{-1}$,

$$\begin{aligned}\widetilde{H}_{\mathrm{LSB},1} = \frac{B}{2}\Bigg[&\frac{F_{sf}^\dagger F_s^\dagger}{a} e^{i\widetilde{\varphi}_s(L)} e^{i\widetilde{\varphi}_{sf}(L)} + \frac{F_s F_{sf}}{a} e^{-i\widetilde{\varphi}_s(L)} e^{-i\widetilde{\varphi}_{sf}(L)} \\ &+ \frac{F_{sf}^\dagger F_s^\dagger}{a} e^{i\widetilde{\varphi}_s(-L)} e^{i\widetilde{\varphi}_{sf}(-L)} + \frac{F_s F_{sf}}{a} e^{-i\widetilde{\varphi}_s(-L)} e^{-i\widetilde{\varphi}_{sf}(-L)} \\ &+ e^{2ik_F L}\Bigg(\frac{F_{sf}^\dagger F_s^\dagger}{a} e^{\frac{i}{2}(\varphi_c(L)+\widetilde{\varphi}_s(L)+\varphi_f(L)+\widetilde{\varphi}_{sf}(L))} e^{-\frac{i}{2}(\varphi_c(-L)-\widetilde{\varphi}_s(-L)+\varphi_f(-L)-\widetilde{\varphi}_{sf}(-L))} \\ &\qquad + \frac{F_s F_{sf}}{a} e^{\frac{i}{2}(\varphi_c(L)-\widetilde{\varphi}_s(L)+\varphi_f(L)-\widetilde{\varphi}_{sf}(L))} e^{-\frac{i}{2}(\varphi_c(-L)+\widetilde{\varphi}_s(-L)+\varphi_f(-L)+\widetilde{\varphi}_{sf}(-L))} \Bigg) \\ &+ e^{-2ik_F L}\Bigg(\frac{F_{sf}^\dagger F_s^\dagger}{a} e^{\frac{i}{2}(\varphi_c(-L)+\widetilde{\varphi}_s(-L)+\varphi_f(-L)+\widetilde{\varphi}_{sf}(-L))} e^{-\frac{i}{2}(\varphi_c(L)-\widetilde{\varphi}_s(L)+\varphi_f(L)-\widetilde{\varphi}_{sf}(L))} \\ &\qquad + \frac{F_s F_{sf}}{a} e^{\frac{i}{2}(\varphi_c(-L)-\widetilde{\varphi}_s(-L)+\varphi_f(-L)-\widetilde{\varphi}_{sf}(-L))} e^{-\frac{i}{2}(\varphi_c(L)+\widetilde{\varphi}_s(L)+\varphi_f(L)+\widetilde{\varphi}_{sf}(L))} \Bigg)\Bigg],\end{aligned} \tag{S46}$$

$$\begin{aligned}\widetilde{H}_{\mathrm{LSB},2} = \frac{B}{2}\Bigg[&\frac{F_{sf} F_s^\dagger}{a} e^{i\widetilde{\varphi}_s(L)} e^{-i\widetilde{\varphi}_{sf}(L)} + \frac{F_s F_{sf}^\dagger}{a} e^{-i\widetilde{\varphi}_s(L)} e^{i\widetilde{\varphi}_{sf}(L)} \\ &+ \frac{F_{sf} F_s^\dagger}{a} e^{i\widetilde{\varphi}_s(-L)} e^{-i\widetilde{\varphi}_{sf}(-L)} + \frac{F_s F_{sf}^\dagger}{a} e^{-i\widetilde{\varphi}_s(-L)} e^{i\widetilde{\varphi}_{sf}(-L)} \\ &+ e^{2ik_F L}\Bigg(- \frac{F_{sf} F_s^\dagger}{a} e^{\frac{i}{2}(\varphi_c(L)+\widetilde{\varphi}_s(L)-\varphi_f(L)-\widetilde{\varphi}_{sf}(L))} e^{-\frac{i}{2}(\varphi_c(-L)-\widetilde{\varphi}_s(-L)-\varphi_f(-L)+\widetilde{\varphi}_{sf}(-L))} \\ &\qquad - \frac{F_s F_{sf}^\dagger}{a} e^{\frac{i}{2}(\varphi_c(L)-\widetilde{\varphi}_s(L)-\varphi_f(L)+\widetilde{\varphi}_{sf}(L))} e^{-\frac{i}{2}(\varphi_c(-L)+\widetilde{\varphi}_s(-L)-\varphi_f(-L)-\widetilde{\varphi}_{sf}(-L))} \Bigg) \\ &+ e^{-2ik_F L}\Bigg(- \frac{F_{sf} F_s^\dagger}{a} e^{\frac{i}{2}(\varphi_c(-L)+\widetilde{\varphi}_s(-L)-\varphi_f(-L)-\widetilde{\varphi}_{sf}(-L))} e^{-\frac{i}{2}(\varphi_c(L)-\widetilde{\varphi}_s(L)-\varphi_f(L)+\widetilde{\varphi}_{sf}(L))} \\ &\qquad - \frac{F_s F_{sf}^\dagger}{a} e^{\frac{i}{2}(\varphi_c(-L)-\widetilde{\varphi}_s(-L)-\varphi_f(-L)+\widetilde{\varphi}_{sf}(-L))} e^{-\frac{i}{2}(\varphi_c(L)+\widetilde{\varphi}_s(L)-\varphi_f(L)-\widetilde{\varphi}_{sf}(L))} \Bigg)\Bigg].\end{aligned} \tag{S47}$$

We calculate $\langle S^x_{\mathrm{imp}}\rangle$ using Eqs. (S45)-(S47). When the local spin symmetry breaking perturbation is applied only to channel 1, only the terms proportional to $e^{2ik_F L}$ and $e^{-2ik_F L}$ in Eq. (S46) contribute in the first order perturbation,

$$\langle S^x_{\mathrm{imp}}\rangle = c \times \frac{B}{L}\left(\frac{1}{\sqrt{\delta\Gamma}} - \frac{1}{\sqrt{\Gamma}}\right) + O(B^2), \tag{S48}$$

where c is a constant. When the local spin symmetry breaking perturbation is applied only to channel 2, we find

$$\langle S^x_{\mathrm{imp}}\rangle = c \times \frac{B}{L}\left(\frac{1}{\sqrt{\delta\Gamma}} + \frac{1}{\sqrt{\Gamma}}\right) + O(B^2), \tag{S49}$$

with the same constant c. Let $\mathcal{N}^{(1)}_{\mathrm{I|E}}$ (resp. $\mathcal{N}^{(2)}_{\mathrm{I|E}}$) be the entanglement negativity between the impurity and the environment when the perturbation is applied to channel 1 (resp. 2). From Eqs. (S48) and (S49), we obtain

$$\frac{1-\mathcal{N}^{(1)}_{\mathrm{I|E}}}{1-\mathcal{N}^{(2)}_{\mathrm{I|E}}} = \frac{1-\sqrt{1-4[c\frac{B}{L}(\frac{1}{\sqrt{\delta\Gamma}}-\frac{1}{\sqrt{\Gamma}})]^2}}{1-\sqrt{1-4[c\frac{B}{L}(\frac{1}{\sqrt{\delta\Gamma}}+\frac{1}{\sqrt{\Gamma}})]^2}} \simeq \left(\frac{\sqrt{\Gamma}-\sqrt{\delta\Gamma}}{\sqrt{\Gamma}+\sqrt{\delta\Gamma}}\right)^2. \tag{S50}$$

Supplementary Note 7. EXPERIMENTAL SETUP

In the main text, we propose to apply a quantum point contact (QPC) on an edge channel at distance L from the metallic dot in the charge Kondo circuit [S17, S18] in Fig. 4a. Below, we show that the QPC causes an LSB, and suggest experimental parameters for detection of the entanglement shells of Kondo clouds.

We first discuss why the QPC causes an LSB. In the circuit, the excess charge of the metallic dot supports an impurity pseudospin 1/2, and electron tunneling between an edge channel and the dot results in pseudospin flip; electron tunneling from the channel to the dot leads to pseudospin flip, saying, from down to up, while tunneling from the dot to the channel results in pseudospin flip from up to down. The QPC causes scattering of an electron on an edge channel, giving rise to asymmetry between down-to-up and up-to-down pseudospin flips. It is because the QPC prevents the electron from tunneling to the dot with the probability determined by the QPC scattering amplitude, reducing the down-to-up pseudospin flip.

We rigorously show this by computing the LDOS of the edge channel at the point where electron tunneling to the dot happens. The Hamiltonian of the edge channel is written as $H_{\text{edge}} + H_{\text{QPC}}$, where $H_{\text{edge}} = i\int_{-\infty}^{\infty} \psi^\dagger(x)\partial\psi(x)\,dx$ describes the edge channel and H_{QPC} describes the QPC,

$$H_{\text{QPC}} = B\psi^\dagger(L)\psi(-L) + B\psi^\dagger(-L)\psi(L). \tag{S51}$$

The field operator $\psi(x)$ annihilates an electron at coordinate x in the chiral edge channel, B is the electron tunneling strength at the QPC, and the tunneling happens between the positions $x = -L$ and $x = L$ on the channel. The Hamiltonian $H_{\text{edge}} + H_{\text{QPC}}$ can be diagonalized as $\sum_\epsilon \epsilon c_\epsilon^\dagger c_\epsilon$ with $c_\epsilon^\dagger = \int_{-\infty}^{\infty} f(x)\psi^\dagger(x)\,dx$. The commutation relation $[H_{\text{edge}} + H_{\text{QPC}}, c_\epsilon^\dagger] = \epsilon c_\epsilon^\dagger$ yields the equation of motion

$$i\partial f(x) + Bf(-L)\delta(x-L) + Bf(L)\delta(x+L) = \epsilon f(x), \tag{S52}$$

and the mode matching method gives

$$f(x) \propto \begin{cases} \exp(-i\epsilon x) & -\infty < x < -L \\ \left(\dfrac{1 - B^2/4}{1 - iB\exp(-2i\epsilon L) + B^2/4}\right)\exp(-i\epsilon x) & -L < x < L \\ \left(\dfrac{1 + iB\exp(2i\epsilon L) + B^2/4}{1 - iB\exp(-2i\epsilon L) + B^2/4}\right)\exp(-i\epsilon x) & L < x < \infty \end{cases}. \tag{S53}$$

The LDOS $\nu(\epsilon)$ of the channel at $x = 0$ (the location at which electron tunneling to the metallic dot happens) is found,

$$\nu(\epsilon) = |f(0)|^2 = \nu_0 \frac{(1 - B^2/4)^2}{(1 - B\sin(2\epsilon L) + B^2/4)^2 + B^2\cos^2(2\epsilon L)} = \nu_0\Big(1 + 2B\sin(2\epsilon L) + O(B^2)\Big), \tag{S54}$$

where ν_0 is a constant. The LDOS $\nu(\epsilon)$ of the edge channel has the same form with the LDOS in Eq. (S12). This shows that the QPC acts as an LSB.

As discussed in the main text, the excess charges of the metallic dot is equivalent with the magnetization of a spin Kondo impurity, and the excess charge can be measured by using a charge detector placed near the dot [S19]. The dependence of the excess charge ΔQ on the distance L provides the information of the spatial distribution of the Kondo cloud, $\rho_n = 1 - \sqrt{1 - 4\langle \Delta Q/e\rangle^2} \simeq 2\langle \Delta Q/e\rangle^2$. We compute the excess charge ΔQ in the Fig. 4b by solving the Hamiltonian $H_{k\text{CK}}^{\text{charge}} + H_{\text{QPC}}$ with the NRG. $H_{k\text{CK}}^{\text{charge}}$ describes the kCK effect in a charge Kondo circuit [S18, S20]

$$H_{k\text{CK}}^{\text{charge}} = \sum_{j=1}^{k} H_j + \sum_{j=1}^{k} J_j(S_{\text{imp}}^+ S_j^- + S_{\text{imp}}^- S_j^+). \tag{S55}$$

H_j describes the j-th chiral edge channel, and H_{QPC} is introduced in Eq. (S51). This Hamiltonian can be solved in the NRG approach with applying the LDOS in Eq. (S54). The excess charge $\langle \Delta Q/e\rangle$ is obtained from the magnetization $\langle S_{\text{imp}}^z\rangle$. The parameters of the NRG calculation are given in Sec. I. The QPC strength B is experimentally obtained from the reflection probability R of the QPC as they are related as $R = B^2/(1 + B^2/4)^2$. We set the QPC strength B such that $R = 0.15$ in our computation.

We suggest experimental parameters of a charge Kondo circuit for detection of the entanglement shells of Kondo clouds. In the realization [S17, S18] of a charge Kondo circuit, the charging energy is about 25 μeV, the Kondo

temperature can be tuned over the range of $0.01\,\mathrm{K} \lesssim T_\mathrm{K} \lesssim 10\,\mathrm{K}$, and the temperature is about 15 mK. This implies that the Kondo cloud length ξ_K is within the range of $0.08\,\mu\mathrm{m} \lesssim \xi_\mathrm{K} \lesssim 80\,\mu\mathrm{m}$. Here, the Fermi velocity is assumed as $v_\mathrm{F} \sim 10^5\,\mathrm{m/s}$ that is typical in integer quantum Hall edge channels. We propose QPC positions L in a range of $3\,\mu\mathrm{m} \lesssim L \lesssim 20\,\mu\mathrm{m}$, which needs to be shorter than the phase coherence length. Variation of L within the range, combined with variation of T_K over the tuning range, allows one to have $0.03 \lesssim L/\xi_\mathrm{K} \lesssim 200$, with which one can measure the dependence of the excess charge on L/ξ_K in Fig. 4 of the main text, hence, the spatial distribution of Kondo clouds in the isotropic kCK effects with $k = 1, 2, 3$. Similarly, in the charge Kondo circuit T^* can be tuned over the range of $10\,\mathrm{mK} \lesssim T^* \lesssim 1\,\mathrm{K}$. It is hence possible to have $\xi^*/\xi_\mathrm{K} = T_\mathrm{K}/T^* \gtrsim 100$. This implies that one can detect the shell structure of Kondo clouds in the anisotropic kCK effects (with $k = 1, 2, 3$) by tuning L/ξ^* and L/ξ_K over a sufficient range. Note that this strategy of varying L/ξ_K was used in a previous experimental report [S21] on observation of a Kondo cloud of the single channel Kondo effect. Sensing the excess charge of $\Delta Q \lesssim 0.01e$, which is necessary to observe crossovers between different entanglement shells of the cloud, is experimentally feasible [S22]. Here e is the electron charge. It would be possible to experimentally measure the power-law decay in Eq. (5), although the power-law behavior is accompanied by the Friedel oscillation, by fine-tuning $2k_F L$ as in an experiment [S21].

Supplementary References

* e-mail: hssim@kaist.ac.kr

[S1] Shim, J., Sim, H.-S. & Lee, S.-S. B. Numerical renormalization group method for entanglement negativity at finite temperature. Phys. Rev. B **97**, 155123 (2018).
[S2] Wilson, K. G. The renormalization group: Critical phenomena and the Kondo problem. Rev. Mod. Phys. **47**, 773 (1975).
[S3] Bulla, R., Costi, T. A. & Pruschke, T. Numerical renormalization group method for quantum impurity systems. Rev. Mod. Phys. **80**, 395 (2008).
[S4] Weichselbaum A. & von Delft, J. Sum-Rule Conserving Spectral Functions from the Numerical Renormaliation Group Phys. Rev. Lett. **99**, 076402 (2007).
[S5] Weichselbaum, A. Tensor networks and the numerical renormalization group. Phys. Rev. B **86**, 245124 (2012).
[S6] Mitchell, A. K., Galpin, M. R., Wilson-Fletcher, S., Logan, D. E. & Bulla, R. Generalized Wilson chain for solving multichannel quantum impurity problems. Phys. Rev. B **89**, 121105(R) (2014).
[S7] Stadler, K. M., Mitchell, A. K., von Delft, J. & Weichselbaum, A. Interleaved numerical renormalization group as an efficient multiband impurity solver. Phys. Rev. B **93**, 235101 (2016).
[S8] Kim, D., Shim, J. & Sim, H.-S. Universal Thermal Entanglement of Multichannel Kondo Effects. Phys. Rev. Lett. **127**, 226801 (2021).
[S9] Ludwig, A. W. W. & Affleck, I. Exact conformal-field-theory results on the multi-channel Kondo effect: Asymptotic three-dimensional space- and time-dependent multi-point and many-particle Green's functions. Nucl. Phys. **B428**, 545 (1994).
[S10] Affleck I. & Ludwig, A. W. W. Critical theory of overscreened Kondo fixed points. Nucl. Phys. **B360**, 641 (1991).
[S11] Andrei, N. & Destri, C. Solution of the Multichannel Kondo Problem. Phys. Rev. Lett. **52**, 364 (1984).
[S12] Emery, V. J. & Kivelson, S. Mapping of the two-channel Kondo problem to a resonant-level model. Phys. Rev. B **46**, 10812 (1992).
[S13] Zaránd G. & von Delft, J. Analytical calculation of the finite-size crossover spectrum of the anisotropic two-channel Kondo model. Phys. Rev. B **61**, 6918 (2000).
[S14] Sela E. & Affleck, I. Nonequilibrium critical behavior for electron tunneling through quantum dots in an Aharonov-Bohm circuit. Phys. Rev. B **79**, 125110 (2009).
[S15] Maldacena J. M. & Ludwig, A. W. W. Majorana fermions, exact mapping between quantum impurity fixed points with four bulk fermion species, and solution of the "Unitarity Puzzle". Nucl. Phys. **B506**, 565 (1997).
[S16] Ye, J. On two channel flavor anisotropic and one channel compactified Kondo models. Nucl. Phys. **B512**, 543 (1998).
[S17] Iftikhar, Z., Jezouin, S., Anthore, A., Gennser, U., Parmentier, F. D., Cavanna, A. & Pierre, F. Two-channel Kondo effect and renormalization flow with macroscopic quantum charge states. Nature (London) **526**, 233 (2015).
[S18] Iftikhar, Z., Anthore, A., Mitchell, A., Parmentier, F. D., Gennser, U., Ouerghi, A., Cavanna, A., Mora, C., Simon, P. & Pierre, F. Tunable quantum criticality and super-ballistic transport in a "charge" Kondo circuit. Science **360**, 1315 (2018).
[S19] Field, M., Smith, C. G., Pepper, M., Ritchie, D. A., Frost, J. E. F., Jones, G. A. C. & Hasko, D. G. Measurements of Coulomb blockade with a noninvasive voltage. Phys. Rev. Lett. **70**, 1311 (1993).
[S20] Mitchell, A., Landau, L. A., Fritz, L. & Sela, E. Universality and Scaling in a Charge Two-Channel Kondo Device. Phys. Rev. Lett. **116**, 157202 (2016).
[S21] Borzenets, I. V., Shim, J., Chen, J. C. H., Ludwig, A., Wieck, A. D., Tarucha, S., Sim, H.-S. & Yamamoto, M. Observation of the Kondo screening cloud. Nature (London) **579**, 210 (2020).
[S22] Pierre, F. Private communication.
